# An integrative approach to dementia care

**DOI:** 10.3389/fragi.2023.1143408

**Published:** 2023-02-16

**Authors:** Alison Warren

**Affiliations:** DAOM, MSHS (Master of science in health sciences), Department of Clinical Research and Leadership, George Washington University School of Medicine and Health Sciences, Washington, DC, United States

**Keywords:** Alzheimer’s disease, dementia, integrated, integrative, quality of life, person-centered, non-pharmacologic

## Abstract

As the aging population continues to increase, Alzheimer’s disease and related dementias are becoming a global health crisis. The burdens experienced by the person living with dementia, their caregivers, healthcare, and society persist unabated. Persons with dementia represent an important population in need of a tenable care plan. Caregivers need the tools with which to properly care for these persons and to mitigate their own stress response. A viable healthcare model utilizing integrated approaches to care for persons with dementia is in overwhelming demand. While much research is focused on a cure, it is equally important to address the difficulties faced by those currently affected. One approach is to incorporate interventions to increase quality of life within the caregiver-patient dyad *via* a comprehensive integrative model. Improving daily life of the persons with dementia, along with their caregivers and loved ones may aid in attenuating the pervasive psychological and physical impacts of this disease. A focus on interventions that provide neural and physical stimulation may facilitate quality of life in this regard. The subjective experience of this disease is challenging to capture. The relationship between neurocognitive stimulation and quality of life is at least, in part, therefore still uncertain. This narrative review aims to explore the efficacy and evidence-base of an integrative approach to dementia care in facilitating optimal cognition and quality of life outcomes. These approaches will be reviewed alongside person-centered care that is fundamental to integrative medicine, including exercise; music; art and creativity; nutrition; psychosocial engagement; memory training; and acupuncture.

## Introduction

Nearly 50 million people globally are affected by Alzheimer’s disease (AD) and other dementias, and this number is projected to double every 20 years (Grande et al., 2020). The annual global economic burden exceeds $1trillion (Grande et al., 2020). Progressive memory decline is the most salient feature of Alzheimer’s disease and related dementias (ADRD), accompanied by declines in language, cognitive functions, activities of daily living, and ultimately leading to death (Leandrou et al., 2020). The behavioral and psychological symptoms of dementia (BPSD) add another layer of negative outcomes for patients and caregivers. BPSD worsens cognitive decline and physical dysfunction in persons with dementia and is associated with increased caregiver burden and distress (Kim et al., 2021). BPSD are often the manifestation of unmet needs in persons who cannot effectively communicate them nor fulfill them autonomously, such as the absence of daytime activities; pain; constipation, loneliness; boredom; or noise (Ferreira et al., 2016; Cunningham et al., 2019). The debilitating nature of the disease affects all aspects of life for the person with dementia, often leading to isolation, loneliness, and decreased quality of life (QoL) (Gulliver et al., 2021).

Considering the pervasiveness of this disease to the individual, family, and society, this major health crisis demands reform to yield more positive outcomes (Olivari et al., 2020). Numerous interventions have been examined individually, with established evidence-base to improve QoL and interactions with caregivers, but very few multidisciplinary programs exist in memory care centers. Drawn from the literature, a multidisciplinary approach can address several care needs, including physical, emotional, and psychosocial avenues with which to optimize care. Building upon the person-centered framework pioneered by Kitwood, integrative medical paradigms can further facilitate the preservation of personhood by avoiding malignant social psychology (i.e., infantilization, disempowerment, ignoring), promoting positive person work (i.e., collaboration, recognition, validation), and attending to the needs of the whole person (Mitchell & Agnelli, 2015). A multifactorial disease such as dementia warrants a multifactorial approach to care. As such, a multidisciplinary integrative approach will be examined and presented to focus on improving QoL for persons with dementia (PWD) and their caregivers.

### Exercise

Exercise confers beneficial effects on the brain in both healthy individuals and persons with various forms of cognitive decline (Morland et al., 2017). Indeed, the positive outcomes of exercise on memory are well-established, and the lack of exercise is likewise a well-documented risk factor for neurological disorders, including dementia (Fernandez et al., 2017). Exercise increases growth factors responsible for plasticity in the brain, including brain-derived neurotrophic factor (BDNF) and insulin-like growth factor 1 (IGF-1), related to the enhanced cognition and antidepressant effects (Lloyd et al., 2017). When done on a regular basis, advantageous effects can be observed at the genomic level *via* promotion of stability of the genome, including remodeling of the BDNF gene and upregulation of BDNF (Lloyd et al., 2017; Rebelo-Marques et al., 2018). Exercise also induces cerebral angiogenesis and vascular endothelial growth factor A, associated with cerebral perfusion and cognitive performance (Morland et al., 2017). Exercise-induced neurogenesis and synaptogenesis take place mainly in the hippocampus, a major area of vulnerability in cognitive decline (Dietrich et al., 2008). Taken together, these are but a few of the main reasons relating to the recommendation of exercise to prevent and delay onset of cognitive decline including Alzheimer’s disease (Erickson et al., 2011; Valenzuela et al., 2020). Erickson and colleagues (2011) conducted a single-blind randomized control trial with 120 older adults randomly assigned to either an aerobic group or stretching control group. They found an increase in hippocampal volume by 2% in the exercise group, thereby reversing age-related volume loss. They also found that the increase in hippocampal volume raised BDNF levels commensurately (Erickson et al., 2011).

Exercise can improve several aspects of Alzheimer’s disease and related dementias *via* its ability to enhance brain plasticity (Fernandes et al., 2017). A cross-sectional study conducted by Sampaio and colleagues (2020) examined the relationship of physical activity in institutionalized persons with dementia and measures of cognition, functional capacity, and QoL. Their findings demonstrated significant positive results across all measures, both globally and *via* caregiver perception ratings, supporting the importance of utilizing exercise as a therapeutic strategy in this patient population (Sampaio et al., 2020). Another recent study by Sampaio and colleagues (2021) examined physical exercise in persons with dementia and perceived benefit by formal caregivers. They utilized a quasi-experimental non-randomized study involving 64 institutionalized older adults with dementia, assigned to either an exercise group or control group, to evaluate functional capacity, BPSD, and QoL perceived by formal caregivers. Their results demonstrated that the exercise group was capable of preserving functional capacity and QoL, and BPSD and caregiver distress were lessened *versus* the control group who showed worsening of symptoms (Sampaio et al., 2020).

A further study of note was conducted by Zhu and colleagues (2018) examined the effect of moderate-intensity aerobic exercise on cognitive function in persons with mild cognitive impairment (MCI). Their single-blind randomized control trial randomized 60 MCI patients to either aerobic dance plus usual care, or a control group with usual care only for 3 months. The aerobic exercise group demonstrated greater improvement in memory, especially episodic memory, and processing speed compared to controls (Zhu et al., 2018).

Despite its promising effects on cognition and mood, many factors remain unknown, including type and frequency of exercise and efficacy in different types and stages of dementia. Balbim and colleagues (2021) conducted a systematic review of 27 RCTs and a meta-analysis of 24 RCTs (2,441 older adults total) to assess exercise interventions in multiple types of dementia, AD, unspecified types, and vascular impairment. They concluded exercises imparts small cognitive improvements in all-cause dementia in older adults, but more research is needed to delineate its effect on different types of dementia (Balbim et al., 2021).

The benefits of exercise are vast. The literature supports several positive outcomes of exercise in persons with dementia and MCI that exceed physical fitness. Moreover, when done in a group setting, exercise of any kind may promote social interaction. Taken together, the literature supports positive cognitive benefits, possible delays in cognitive decline, decreased BPSD, and increased QoL. More robust research is needed to uncover the precise effects of exercise in different types and stages of impairment, but even small improvements may confer great benefit for those at risk or experiencing cognitive decline. This support merits inclusion in an integrative approach to dementia care.

### Music listening

Persons with AD and related dementias often exhibit intact musical processing (Deason et al., 2019). As components of musical memory and processing are spared in person with AD, music, including musical mnemonics may serve to improve memory, alleviate some symptoms of dementia, and enhance self-consciousness (Arroyo-Anlló et al., 2013; Deason et al., 2019).This preferential sparing may allow for recognition, heightened arousal, improved attention and memory, and reduced BPSD (Simmons-Stern et al., 2010; Buller et al., 2019). Studies have demonstrated that persons with AD demonstrate better accuracy at recognition of sung lyrics than spoken lyrics (Simmons-Stern et al., 2010; Buller et al., 2019). Moreover, numerous anecdotal and controlled group studies have exhibited remarkable lucidity in persons with severe AD, described as an “awakening”, and benefits of autobiographical memory recall and cognitive performance tests in response to music listening (Simmons-Stern et al., 2010).

Personalized music playlists are one avenue with which to deliver the benefits of music to persons with ADRD. Murphy and colleagues (2018) examined the effectiveness and feasibility of implementing personalized music listening for assisted living residents with dementia. They assessed the dimensions of reach, effectiveness, adoption, implementation, and maintenance in an assisted living facility over 8 months. Positive evidence across all dimensions indicated personalized music listening to be a low-cost meaningful intervention for persons with dementia (Murphy et al., 2018).

Later, “The Roth Project—Music and Memory” program was implemented to assess the effect of individualized music on neuropsychiatric symptoms of dementia (Buller et al., 2019). 79 caregivers assessed satisfaction and perception of impact on mood and behavior. 99% reported satisfaction with the program and 94% reported perceived positive effects, including enjoyment, improved overall happiness, decreased anxiety and depression, and increased positive emotional expression (Buller et al., 2019).

Considering the multitude of benefits of music, especially personalized music, coupled with the low cost and ease of implementation, it should be an integral part of an integrative approach to dementia care.

### Nutrition and eating

Nutrition occupies a critical position in both the prevention and management of dementia. It also serves an important role in the onset and prognosis of dementia (Brockdorf and Morley, 2021). As such, nutritional assessment and intervention provides rich potential when nested within a comprehensive integrative approach to dementia care (Cesari et al., 2021). When overlooked, a vicious cycle may present as cognitive decline affects nutritional status, and malnutrition in turn worsens cognitive decline (Cesari et al., 2021). Indeed, even the most well-designed pharmaceutical will fail in the face of unmet needs that are fundamental to healthy living, such as social interaction, physical activity, and a healthy diet (Cesari et al., 2021). The World Health Organization has recognized the problem of malnutrition in this population and recently developed the Integrated Care for Older People (ICOPE) program to promote functional ability and intervention steps to manage declines in older age, including malnourishment and frailty (Won et al., 2021).

The brain “requires a disproportionate amount of energy compared to its mass” (Brockdorf and Morley, 2021, p. 590), particularly in the form of glucose to maintain normal function. Neuronal glucose metabolism declines in the aging brain, resulting in an ATP deficit, decreased NAD, and restricted glucose access (Blaszczyk, 2020). Brain insulin resistance is one of many pathophysiological factors in AD and dementia (Hallschmid, 2021). Vigilant attention to proper glucose maintenance for the brain and the body may therefore optimize health and daily activity performance.

In this vein, insulin and glucose maintenance are also imperative in the periphery. Malnutrition, including a lack of energy and micronutrients, can accelerate the progression and manifestations of dementia (Tangvik et al., 2021). Western diets, rife with processed foods, meat, saturated fatty acids, trans-fats, and high sugar foods and drinks have been associated with insulin resistance and several disease states, as well as smaller hippocampi and greater cognitive decline (Brockdorf and Morley, 2021). These foods are also associated with insulin resistance and type II diabetes, which itself also a risk factor for dementia (Brockdorf and Morley, 2021). The Mediterranean diet, characterized by fruits, vegetables, legumes, nuts, olive oil, unrefined cereals, fish, moderate wine, and low intake of dairy, meat, poultry, and saturated fat is associated with lower incidences of several diseases and inversely associated with dementia (Andreu-Reinón et al., 2021). Those who eat a Mediterranean diet have lower rates of chronic illness, cognitive decline, and AD, as well as better cognition and structural connectivity (Brockdorf and Morley, 2021). The MIND diet, a combination of the Mediterranean diet and the Dietary Approaches to Stop Hypertension (DASH) is also associated with slower cognitive decline and decreased risk of AD (Dhana et al., 2021). Several micronutrients derived from whole foods are likewise noteworthy in consideration of dietary intervention for person with dementia. In a randomized, double-blind, placebo-controlled trial, older adults with cognitive decline who were given daily blueberry supplementation for 16 weeks exhibited increased blood oxygen level dependent (BOLD) activation in several brain areas during working memory challenge, although working memory enhancement was not observed (Boespflug et al., 2017). Micronutrients essential to brain function include, but are not limited to choline; amino acids, copper; selenium; manganese; zinc; and antioxidants (Brockdorf & Morley, 2021). While still under debate, there is some evidence to suggest possible benefit of Acetyl-L-Carnitine supplementation *via* gut—liver—brain axis regulation for persons with dementia (Pennisi et al., 2020). When appropriate, nutritional supplementation may be appropriate to facilitate intake of energy and protein in persons with dementia (Tangvik et al., 2021), Many nutraceuticals are of interest (i.e., Phosphatidylserine, Ginko biloba, Huperzine, A, Vinpocetine, Omega 3 oils etc.) (Sierpina et al., 2005), but still inconclusive. Periodic nutritional screening may likewise prove beneficial in this regard.

The ways in which we psychologically perceive, sense, and physically take in food also serve a critical role in nutrient absorption. Several factors affect eating habits and therefore nutritional status during the course of cognitive decline. Some considerations include, but are not limited to, change in olfactory and taste discrimination; impaired decision-making abilities; inability to identify food preferences; poor food quality; social isolation; depression (Cesari et al., 2021); inability to recognize food or understand what to do with it; and inability express preferences or desires (Hannon, 2018); rigid mealtimes; and excessively restrictive diets (Sadarangani et al., 2021). Indeed, persons with dementia often experience problems with nutrition for a multitude of reasons (Tangvik et al., 2021). Loss of appetite, loss of eating skills, difficulty communicating discomfort, including hunger, can all negatively affect food intake resulting in malnourishment, weight loss, and frailty (Tangvik et al., 2021). The consequential decline in cognition and muscle wasting contributes to loss of independence and functional declines that increase risk of morbidity and mortality (Tangvik et al., 2021). What’s more, persons with dementia who are malnourished score higher on dementia behavior disturbance parameters and exhibit BPSD, underscoring a bidirectional relationship (Kimura et al., 2019), which can lead to increased internal stress and caregiver burnout. A study by Kimura and colleagues (2019) found that nutritional status was significantly correlated with specific BPSD including verbal aggressiveness, emotional disinhibition, apathy, and memory impairment.

It is of paramount import to not only optimize nutrition, but also emphasize all possible qualities of autonomy that can be preserved in persons with dementia. In this regard, mealtimes and food choices represent one avenue with which to provide a “sense of meaning, order, and structure” (Hannon, 2018; p. 259). Cultural competency is an additional aspect of person-centered nutritional care to consider in persons with dementia. Sadarangani and colleagues (2021) conducted a qualitative analysis of the delivery of person-centered nutrition to Asian Americans with dementia in an adult day care setting. They found that providing opportunities for peer relationships, culturally tailored meals, celebrations, and consistent staff assistance with feeding greatly benefited persons with dementia (Sadarangani et al., 2021).

### Facilitating creative, cognitive, and social stimulation

Numerous positive outcomes can be gained through psychosocial activities that affect QoL. The literature supports the benefits of incorporating art activities in dementia care, including increases in positive affect, QoL measures, social interaction and support, self-esteem, and confidence (Robertson and McCall, 2020). Indeed, the potential psychosocial and sensory stimulatory benefits are increasingly recognized by various specialties of medical professions (Guseva, 2019). Art-based activities provide opportunities for intellectual stimulation with positive impacts on cognition, communication, attention, concentration, and personhood (Robertson and McCall, 2020). Furthermore, creative therapies (i.e., art, music, storytelling, dance) generate opportunities for reminiscence and self-expression leading to enhanced well-being, improved memory, social and cognitive functioning, and decreased anxiety and cognitive decline (Foti and Ghaul, 2019). Art-based activities therefore foster creativity, self-expression, communication, including nonverbal communication, and decrease the isolation and loneliness so commonly seen in person with dementia (Robertson and McCall, 2020). In this way, art activities hold therapeutic value by serving as “a vehicle for nonverbal communication that gives people with dementia a means to be understood and to have their emotions validated by others” (Guseva, 2019, p. 46).

In Japan, short-term intensive rehabilitation programs for persons with dementia utilize evidenced-based interventions such as reminiscence therapy, reality orientation, memory rehabilitation, physical exercise, and occupation therapy, all shown to improve cognitive function and reduce BPSD (Tanaka et al., 2021). In consideration of those who are in long-term care facilities, Tanaka and colleagues (2021) examined the effects of a group-based physical and cognitive intervention on social activity and QoL for elderly people with dementia. They conducted a quasi-randomized controlled trial of 31 participants with dementia in a geriatric health care facility that entailed an 8-week exercise and cognitive stimulation program in the intervention group and found the intervention group demonstrated increased social activity, QoL, and helping behavior compared to controls (Tanaka et al., 2021).

As part of recent movements to enhance physical, psychological, and social engagement, dance programs are being increasingly implemented in long-term care centers (Kontos et al., 2021). Kontos and colleagues (2021) conducted a sequential multiphase qualitative study involving 67 persons with dementia and 15 family caregivers using the Sharing Dance Seniors program, which remotely streams weekly dance sessions. They found this intervention increased playfulness and sociability of participants, underscoring the importance of social support, play, and imagination in persons with dementia (Kontos et al., 2021).

### Cognitive behavioral therapy

While there are numerous mind-body interventions to facilitate healthy mindsets and behaviors, cognitive behavioral therapy (CBT) is a technique that can be adapted to persons with dementia as well as caregivers, and can be delivered in multiple formats (i.e., individual, group, telehealth). For PWD and MCI, CBT can be modified to include learning adaptations and dementia-specific strategies, such as increased frequency and shorter duration to optimize outcomes (Charlesworth et al., 2015; Jin et al., 2021).

CBT has garnered significant attention in recent years for its ability to address depression and anxiety in older adults (Koder, 2018; Fossey et al., 2021). This psychological approach is aimed at reducing mental health symptoms by addressing the interaction between one’s thoughts, feelings, and behaviors (Fossey et al., 2021). It is a goal-oriented approach aimed at reframing an individual’s thought processes (Jin et al., 2021). In this way, CBT may facilitate amelioration of depression, anxiety, and BPSD in persons with dementia, thereby avoiding inappropriate sedation (Koder, 2018). What’s more, evidence suggests that it may help with insomnia (Jin et al., 2021), a common symptom in PWD that, like persons without dementia, can have a variety of deleterious effects on health and increase the risk of injury. Nonpharmacological interventions such as CBT that have demonstrated efficacy in improving the quality of life in PWD, as well as their caregivers, are of paramount importance to include in an integrative care model.

### Environmental design

The environment within which one resides is an important factor to consider in mental and physical health (Chaudhury et al., 2018), and lends greatly to the distinction between existing and thriving. Integrative care models emphasize optimal healing environments, in which the physical spaces support psychological, social, spiritual, behavioral, and physical well-being (Rakel and Minichiello, 2023). Optimal healing environments are intended to surpass mere aesthetics by also creating environments that support intrapersonal, interpersonal, and positive lifestyle engagement (Rakel and Minichiello, 2023). Utilizing aspects of nature, fresh air, art, music, and color can be of great benefit in this regard, in both inpatient and outpatient care facilities.

Nursing homes and long-term memory care centers, ostensibly dedicated to caring for loved ones, are still often perceived as cold and sterile (Thomas, 1996). Adopting minor changes to enhance the physical environment, however, can have a profound effect on one’s psychological and physical state of health. William Thomas has been among the pioneers who have created a model of a “deinstitutionalized nursing home” through his Green House concept (Rabig et al., 2006). Patterned after a residential home, a Green House is a self-contained home for a small group that blends privacy and autonomy with a de-medicalized social community with fellow residents and staff, such that willing participants engage in household chores and daily activities (i.e., sharing meals, decision making, gardening) (Thomas, 1996; Rabig et al., 2006). This model, therefore, promotes dignity along with a sense of purpose and meaning, values which when lacking, erode innumerable aspects of the self.

In addition to the physical and cultural community of nursing homes, incorporating aspects of nature into the environment has also demonstrated value. Healthcare facilities that include gardens provide therapeutic benefits, such as visually stimulating pleasant views of nature that create a calming effect; an avenue for social interaction as well as privacy; stress reduction; increase mobility; and improved feelings of loneliness and depression (Uwajeh et al., 2019). Simply having this space available provides positive sensory and psychological stimulation. When PWD actively engage with these spaces, (i.e., weeding, planting, cultivating), horticulture provides a kind of therapy that has been associated with multiple positive outcomes, including social engagement, improved mood, cognition, and motor function, as well as new memory formation (Scott et al., 2022). Spaces that include nature and gardens are a low-cost high-benefit strategy to implement in long-term care facilities that would aid both patients and employees.

### Acupuncture

Traditional Chinese Medicine (TCM) has been used in China for thousands of years. While TCM encompasses a variety of techniques (food therapy, acupuncture, herbal medicine, moxibustion, guasha, cupping, taichi, qi gong), this review will focus on acupuncture. In one of the largest meta-analyses involving acupuncture for chronic pain, 29 randomized controlled trials involving at total 17, 922 participants found acupuncture to be superior to placebo (Vickers et al., 2012). Numerous hospitals and government agencies, including the U.S. Veteran’s Administration utilize acupuncture, and consider this study to be one of the most supportive (Kaptchuk, 2020). Several challenges are inherent in acupuncture research. It is a sophisticated system with differing neurological effects based on acupoint specificity and provider manipulation. Nonetheless, its high safety profile and ability to affect the nervous system and immune system merit inclusion in an integrative approach to dementia care, with acknowledgement that further research validation is warranted.

The WHO recommends acupuncture for over 90 conditions (Lan et al., 2020). Brain imaging technologies including fMRI and PET have been used to assess the dynamic brain mechanisms to acupuncture and its utility in several disorders (Yu et al., 2019). In relation to neurocognitive decline specifically, acupuncture is thought to regulate neurotransmitters, decrease oxidative stress, promote removal of free radicals, inhibit neuronal apoptosis, decrease neuroinflammation and microglial overactivation, activate of hippocampal protein kinases, and inhibit of the expression of microvessel tau protein (He et al., 2021). Additionally, acupuncture stimulation to specific points may affect the functional activity of large-scale brain networks, which may account for improvements in cognitive function in persons with AD (Ji et al., 2021). Systematic reviews suggest the potential for acupuncture to serve as a safe and effective intervention for cognitive decline to improve cognitive function (Lan et al., 2020; He et al., 2021). Acupuncture is widely used in persons with AD to ameliorate neuropsychiatric symptoms.^23^ A study in 2014 by Wang et al. used fMRI to evaluate the effects of acupuncture on hippocampal connectivity in patients with AD. A total of 28 participants were evaluated before and after acupuncture administration and found that 14 AD patients demonstrated increased connectivity in the hippocampi following acupuncture treatment Wang et al., 2014).

A recent overview of 13 systematic reviews with meta-analyses was conducted by Ma and colleagues (2021) including 137 randomized controlled trials with a total of 9,012 participants. They found that acupuncture had beneficial effects on effectiveness, cognitive ability, and activities of daily living for persons with dementia (Ma et al., 2021). Further analysis concluded that acupuncture may have superior efficacy in vascular dementia *versus* AD (Ma et al., 2021).

A study by Su and colleagues (2021) examined the effectiveness and safety of acupuncture on vascular cognitive impairment by reviewing 48 RCTs involving 3,778 patients. They found that when compared to western medicine, acupuncture was more beneficial for global cognitive function, and there was no difference in adverse effects between the acupuncture and control groups. Like many acupuncture studies, low methodological quality and heterogeneity are concerns that must be addressed in future studies. However, their analysis suggests that acupuncture may have a positive impact on cognition and daily performance with few side effects (Su et al., 2021).

### Integrative treatment programs

Examined individually, the above modalities seem to hold significant value, but in the context of daily life, utilizing only one or two interventions may not confer the level of benefit needed to alter quality of life in those with cognitive impairment. Combining these positive effects, however, such that a treatment intervention may become adapted to lifestyle changes, may contribute more meaningful change overall, especially in those who are at the earlier stages of impairment.

The importance of a multimodal integrative treatment approach is gaining recognition. On an outpatient basis, all patients who seek care from an Integrative Medicine practitioner will receive an individualized approach tailored to their unique needs and health goals. It is common for this to include multiple lifestyle recommendations and adjunctive therapies, such as acupuncture, yoga, and mind-body techniques (i.e., meditation, mindfulness, taichi, CBT). Utilizing integrative medicine in long-term care facilities is still a nascent field, but one in concert with person-centered, wellness-driven care.

Ahn and Hyun (2019) conducted a pre and post evaluation of an integrative medicine program in community dwelling elderly without a diagnosis of dementia in Korea. This 8-week program included acupuncture, moxibustion, physical activity, meditation, laughter therapy, and music therapy. While MMSE scores did not increase, as expected from cognitively intact participants, Trail Making Test scores improved significantly, depression scores decreased, and preventative behaviors for dementia increased significantly. These results suggest that an integrative program may be beneficial for executive function and preventative lifestyle behaviors (Ahn and Hyun, 2019).

In the United States, one program that merits recognition is The Mayo Clinic’s Healthy Action to Benefit Independence and Thinking (HABIT) program, which encompasses 50 h (10 days) of cognitive rehabilitation and wellness activities for persons with MCI and their care partners. This group-based program utilizes memory compensation training, yoga, support groups, and wellness interventions to promote healthy behaviors. Evaluation of the program demonstrated an ability to learn memory support system despite the presence of MCI, improved memory-related activities of daily living, and increases in measures of self-efficacy and quality of life (Locke et al., 2021).

Integrative medicine programs for cognitive impairment are not yet as widespread as needed by the demand of increasing prevalence of dementia. Discerning the elements that are efficacious and those lacking efficacy can guide institutions toward adoption of similar models. While more data is needed to identify the most optimal program, the available literature suggests that an integrative medical program may provide ect significant improvement in the quality of life and daily behaviors of persons with cognitive impairment, as well as their caregivers.

## Discussion

Integrative medicine embraces a whole person lifestyle approach to care for the patient. This incorporates both their internal and external environments, including the family unit. It is aimed at examining health and pathology through a systems biology approach, utilizing the relationships between body systems rather than separating physiology into silos. Psychological, physical, and lifestyle factors are therefore evaluated for their effects on neuroinflammation, as well as their effects on one’s subjective internal experience. As such, the dilution of limiting medical advice to simplistic individual recommendations to promote brain health would be a disservice. It follows then that complexity of brain health cannot be reduced to a simple increase in blueberry and fish consumption, or the addition of turmeric supplements or a multivitamin. These changes alone are likely insufficient to create meaningful change, despite their ability to temporarily increase neural activation. Biological and environmental contexts are critical components to consider as well.

Moving beyond isolated interventions that are only offered in intervals may provide the opportunity for these behaviors and interventions to culminate into a significant impact within the dyad. A cohesive integrated model that addresses multiple physiological and psychological factors is necessary to effect change, rather than the more common piecemeal inclusion of interventions. As part of a long-term lifestyle and daily care plan, it is possible that these small interventions may have an additive effect in this regard. Reviewing the evidence-base for interventions individually helps to establish validity, but it is correspondingly important to recognize the potential of combining approaches to address multiple factors simultaneously.

One such critical factor that cannot be overstated in is a person-centered approach when incorporating integrative interventions. A shift in focus to prioritize the strengths retained in persons with ADRD is essential to promote and maintain QoL. This change in perspective and behavior may attenuate the inevitable burden experienced by persons with dementia, their caregivers, and their loved ones to some extent. In persons experiencing cognitive decline, even small advances can create meaningful impacts. While the usage of integrative interventions is increasing, a comprehensive model that can be easily adopted by care facilities is needed.

What’s more, a successful program in which participants enjoy their time at a care center, would perhaps result in less guilt will be experienced by the caregiver. This in turn could promote opportunities for the caregiver to practice self-care without the guilt of being away from their loved one. Moreover, less burden and stress within this dyad may allow persons with dementia to remain at home with family for as long as possible, which for many, is an ultimate goal to improve quality of life. More research is needed to determine the effect of an integrative care program on delayed admittance to long-term care facilities. Finally, an additional layer to consider for an integrative care program is to implement tasks for persons with dementia to promote autonomy and purpose. There are many other integrative modalities worth considering that exceeded the scope of this review, including but not limited to meditation, mindfulness, mindfulness-based stress reduction, tai chi, qi gong, yoga, and more.

An integrative approach to a prodigiously multifaceted disease may thereby serve to slow decline, reduce stress, and most importantly, enhance interpersonal communication during this difficult journey. A care model that is rooted in person-centered care, prioritizes non-pharmacological approaches into daily life, and integrates evidence-based, quality of life driven interventions, is not only sorely needed, but owed to this patient population. Integrative medicine excels at this in outpatient care but is perhaps underutilized in long-term in-patient care. This incorporation of an integrative care model may aid in shifting the narrative of dementia from one of loss and crisis, to one of maintained dignity and care.
